# Pavlov’s experiment-inspired optical neural networks based on dual-color fluorescence switching effect

**DOI:** 10.1093/nsr/nwag029

**Published:** 2026-01-19

**Authors:** Songrui Wei, Kunbin Huang, Dingchen Wang, Shangcheng Yang, Haiyan Huang, Xiao Tang, Yanqi Ge, Bowen Du, Zhi Chen, Zhongrui Wang, Shuqing Chen, Dror Fixler, Dianyuan Fan, Han Zhang

**Affiliations:** College of Physics and Optoelectronic Engineering, State Key Laboratory of Radio Frequency Heterogeneous Integration, Shenzhen University, Shenzhen 518060, China; College of Physics and Optoelectronic Engineering, State Key Laboratory of Radio Frequency Heterogeneous Integration, Shenzhen University, Shenzhen 518060, China; Department of Electrical and Electronic Engineering, University of Hong Kong, Hong Kong 999077, China; College of Physics and Optoelectronic Engineering, State Key Laboratory of Radio Frequency Heterogeneous Integration, Shenzhen University, Shenzhen 518060, China; College of Physics and Optoelectronic Engineering, State Key Laboratory of Radio Frequency Heterogeneous Integration, Shenzhen University, Shenzhen 518060, China; College of Physics and Optoelectronic Engineering, State Key Laboratory of Radio Frequency Heterogeneous Integration, Shenzhen University, Shenzhen 518060, China; College of Physics and Optoelectronic Engineering, State Key Laboratory of Radio Frequency Heterogeneous Integration, Shenzhen University, Shenzhen 518060, China; College of Physics and Optoelectronic Engineering, State Key Laboratory of Radio Frequency Heterogeneous Integration, Shenzhen University, Shenzhen 518060, China; College of Physics and Optoelectronic Engineering, State Key Laboratory of Radio Frequency Heterogeneous Integration, Shenzhen University, Shenzhen 518060, China; School of Microelectronics, Southern University of Science and Technology, Shenzhen 518055, China; College of Physics and Optoelectronic Engineering, State Key Laboratory of Radio Frequency Heterogeneous Integration, Shenzhen University, Shenzhen 518060, China; Department of Engineering, Bar-Illan University, Ramat Gan 5290002, Israel; College of Physics and Optoelectronic Engineering, State Key Laboratory of Radio Frequency Heterogeneous Integration, Shenzhen University, Shenzhen 518060, China; College of Physics and Optoelectronic Engineering, State Key Laboratory of Radio Frequency Heterogeneous Integration, Shenzhen University, Shenzhen 518060, China

**Keywords:** optical computing, optical neural network, associative learning, Pavlov’s experiment, dual-color photoinitiator

## Abstract

Cutting-edge optical neural networks are often still trained by backpropagation, which is computationally intensive and originally for conventional artificial neural networks. Inspired by Pavlov’s experiment, we drew upon the principles of biological memory to establish an associative learning framework for training optical neural networks that mimics the mechanisms of associative learning and synaptic plasticity using dual-wavelength stimuli (i.e. ultraviolet and visible light) on a dual-color photoinitiator resin. Sequential light irradiation was shown to induce fluorescence switching and encode associative memory in the resin, which can serve as a physical substrate for an optical neural network. In optical experiments, the established framework was applied to pattern recognition of the letters ‘N,’ ‘V,’ and ‘Z.’ Simulations were conducted that extended its application to the recognition of handwritten digits. Compared to the current mainstream ‘bottom-up’ optical neural network fabrication approach that requires ‘weight calculation followed by hardware implementation’, this work presents a novel ‘top-down’ *in-situ* training methodology that eliminates the need for weight computation. The proposed method holds significant implications for large-scale, low-cost, and rapid fabrication of optical neural networks intended for edge computing applications. This study bridges biological learning principles with optical neural networks to provide a foundation for next-generation adaptive and scalable artificial intelligence systems.

## Teaser

Inspired by Pavlov’s experiment and associative learning theory, we realized in-situ hardware training of optical neural network with dual-color photoinitiator materials.

## INTRODUCTION

The mechanisms underpinning intelligence and memory remain a cornerstone of scientific inquiry and bridging disciplines from biology to artificial intelligence [1–5]. Memory is a foundational element of intelligence and has been extensively investigated at both the macroscopic and microscopic levels [3–12]. Pavlov’s seminal experiments provided a macroscopic framework for understanding associative memory [13]. At the microscopic level, Hebb and Konorski proposed complementary theories linking memory to synaptic plasticity (i.e. a change in morphology of the connections between neurons) [2,12,14]. These theories indicate that memory is encoded by both biochemical and structural mechanisms.

**Figure 1.**
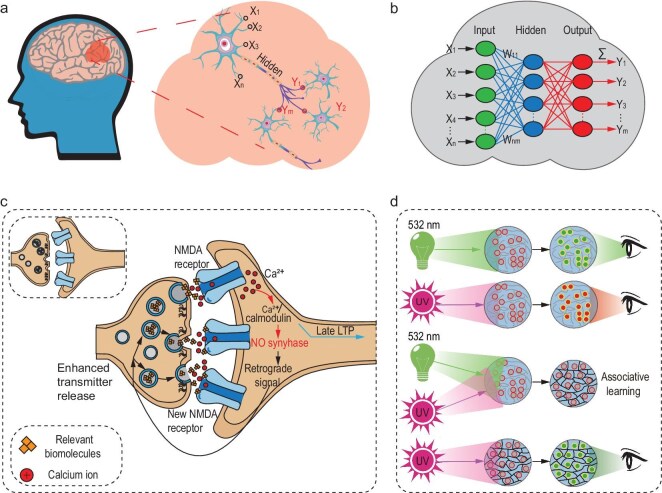
Relationships between biological intelligence and artificial intelligence. (a) Nervous system at the cell level. (b) Architecture of an ANN. (c) Synaptic plasticity in a biological synapse: two materials work together to change the morphology of the synapse. (d) Synaptic plasticity in an optical synapse: two wavelengths of light work together to change the weight of an ONN via associative learning.

The biological principles of neural function have inspired the development of artificial neural networks (ANNs), which have revolutionized the field of artificial intelligence [15–17]. However, ANNs are predominantly trained by backpropagation (BP), which is computationally intensive and energy-inefficient and thus limits their scalability and application in resource-constrained environments [7–9]. Emerging hardware paradigms such as optical neural networks (ONNs) offer a promising solution owing to their inherent parallelism, low energy consumption, and in-memory computing capabilities [18–25]. Despite these advantages, however, ONNs still rely on BP algorithms implemented on traditional electronic hardware, which limits their potential. These constraints have prompted researchers to ask if further inspiration be drawn from biological processes to train ANNs in a more efficient and scalable manner. Training an ANN involves the optimization of weights, which stores information analogous to how memory works in biological systems. If the principles of memory formation are revisited, parallels can be observed between the macroscopic observations of Pavlov [11] and the microscopic mechanisms proposed by Hebb and Konorski [14]: both frameworks underscore the importance of dual signals, simultaneous activation, and repetitive stimulation in the learning process. These insights led to a new paradigm for ANN training called associative learning [26–31], which fundamentally deviates from BP by emulating biological learning processes. Figure 1 shows how biological intelligence is emulated by artificial intelligence. Figure 1a shows a biological neural network, which is emulated by an ANN as shown in Fig. 1b. A biological neural network stores memory in a biological synapse via synaptic plasticity, as shown in Fig. 1c. In an ONN, a biological synapse is emulated by an optical synapse, which imitates synaptic plasticity by dual-color fluorescence switching as shown in Fig. 1d.

In this study, we established an associative learning framework for ONNs inspired by biological memory and demonstrated its feasibility through a series of optical experiments and computational simulations. First, we investigated the fluorescence switching in a dual-color photoinitiator (DCPI) resin [32,33] using an approach analogous to Pavlov’s experiment. This resin switches fluorescence according to photochemical principles and thus provides a physical substrate for emulating associative learning [34]. We analyzed the photophysical mechanisms underlying this phenomenon and linked the fluorescence response to the temporal sequence and pairing of stimuli. We built on this foundation to design an optical experiment where we applied the DCPI resin to image recognition. Associative learning was leveraged by employing dual optical signals to encode associative relationships with weights representing learned connections between inputs and outputs in an emulation of synaptic plasticity. As a proof of concept, the established framework was applied to recognize the letters ‘N,’ ‘V,’ and ‘Z.’ Simulations were performed to extend the established framework to large-scale tasks including handwritten digit recognition to showcase its scalability and robustness.

## KEY CHEMICAL REACTIONS AND THE COMPARISON WITH THE BIOLOGICAL SYSTEM

### Key chemical reactions

**Figure 2.**
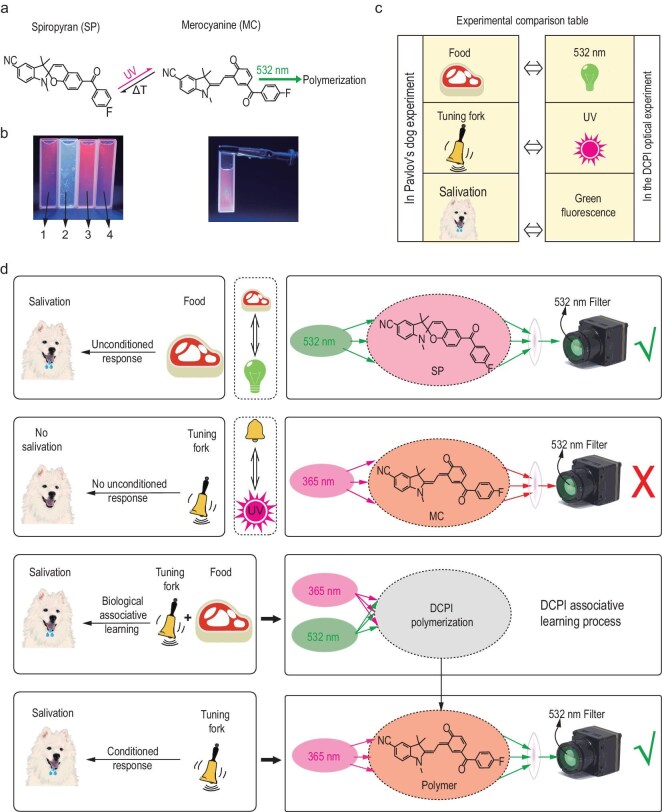
Dual-color fluorescence switching mechanism. (a) Polymerization dynamics of the DCPI resin. (b) Experimental observations of the fluorescence responses to UV and visible light irradiation. (c) A comparison table between the terms in Pavlov’s experiment and our dual-color fluorescence switching experiment. (d) Analogy between Pavlov’s experiment and the stimulus–response association learned by the resin.

Figure 2a shows the dual-color fluorescence switching mechanism of the DCPI resin, which involves a two-step reaction sequence [32]. First, spiropyran (SP) undergoes a photochemical transformation into merocyanine (MC) upon ultraviolet (UV) irradiation. This reaction is reversible. In the absence of UV irradiation, MC gradually reverts to SP at room temperature because of the thermally induced back-reactions. MC emits red fluorescence under UV irradiation. Second, MC is polymerized under visible light irradiation, which in this study was applied by a 532-nm green laser. This step is irreversible. The polymerized product emits green fluorescence under UV irradiation. In this work, DCPI and SP are synonymous terms referring to the same material, where SP represents its chemical nomenclature while DCPI denotes its functional designation. Additionally, both SP and MC represent material classes rather than specific materials.

The left panel of Fig. 2b shows four cuvettes demonstrating this photochemical behavior. Cuvette 1 is only subjected to UV light. So, only the first step of the reaction is activated. The SP transforms to MC and emits red light under UV irradiation. Cuvette 2 is projected by UV light and visible light in sequence. Thus, the whole reaction is activated. The polymer of MC emits green light under UV irradiation. Cuvette 3 is only projected by visible light while cuvette 4 is projected both by visible light and UV light in sequence. Both of them do not meet the reaction conditions and no MC polymer is produced. So, the fluorescent properties of cuvettes 3 and 4 are similar to cuvette 1 under UV irradiation. For all four cuvettes, the UV irradiation intensity decreased from top to bottom, and the intensity of both red and green fluorescence exhibited a direct positive correlation with the intensity of the UV irradiation, which indicates that the fluorescence response is intensity-dependent. The right panel of Fig. 2b shows a cuvette that was uniformly exposed to UV irradiation. The polymer emits a green fluorescence while the surrounding MC emits a red fluorescence. This result highlights the distinct and spatially resolved fluorescence responses of the two materials under UV irradiation.

### Comparison with the biological system

These observations were used to establish a stimulus–response association analogous to Pavlov’s experiment, as illustrated in Fig. 2d. Visible light irradiation is analogous to food, UV irradiation is analogous to the bell ringing, and the green fluorescence is analogous to the salivation of the dog (Fig. 2c). When the DCPI resin is irradiated with visible light, part of the incident light is scattered, and a green fluorescence is emitted. This is analogous to food triggering salivation of the dog (i.e. unconditioned response). Conversely, irradiating the resin with UV light results in a red fluorescence but no green fluorescence. This is analogous to ringing the bell not causing any salivation because the dog has not been conditioned (i.e. neutral response). When the DCPI resin is exposed sequentially to UV irradiation and then visible light, it undergoes polymerization. Subsequent UV irradiation results in a green fluorescence. This sequential exposure associates the visible light and UV irradiation, which is analogous to conditioning the dog to salivate in response to the bell (i.e. conditioned response). Thus, a stimulus–response association is learned.

## RESULTS

In this work, the synthesis method for DCPI was adapted from a referenced literature [32]. We have summarized the synthetic procedure in Section 1 of the Methods and provided detailed optical experimental protocols for fluorescence characterization of DCPI. The optical experimental setup and testing methodology for constructing and validating the optical neural network using DCPI are illustrated in Figs S1–S3, including schematic diagrams and experimental photographs. To confirm material consistency with the referenced work, we performed nuclear magnetic resonance (NMR) spectroscopy characterization and compared the results with those reported in the literature, as shown in Figs S4–S6. Furthermore, we conducted stability tests of the material under illumination, with corresponding data presented in Figs S7–S10.

### Characterization of fluorescence switching

First, we quantitatively characterize the physical processes experienced by the four cuvettes in Fig. 2a, thereby verifying that visible light accelerates the polymerization of MC, causing them to emit green fluorescence earlier under UV irradiation. The significance of timing lies in the fact that UV light alone can also slowly induce polymerization. Therefore, comparison with the Pavlovian experiment is only valid within a specific time window, a period during which UV irradiation alone has not yet triggered green fluorescence, while irradiation with UV followed by visible light can induce green fluorescence. In subsequent reasoning, all sampling of results is conducted within this time window. We define the time point at which green fluorescence intensity exceeds red fluorescence intensity under UV irradiation as the ‘fluorescence switching time’ and use this as a parameter to compare the processes experienced by the four cuvettes shown in Fig. 2a. It is worth mentioning that the issue of UV light slowly inducing polymerization is an area worthy of improvement in this experiment. The referenced work utilizing this effect for 3D printing also adopted various measures to mitigate this issue [32].

**Figure 3.**
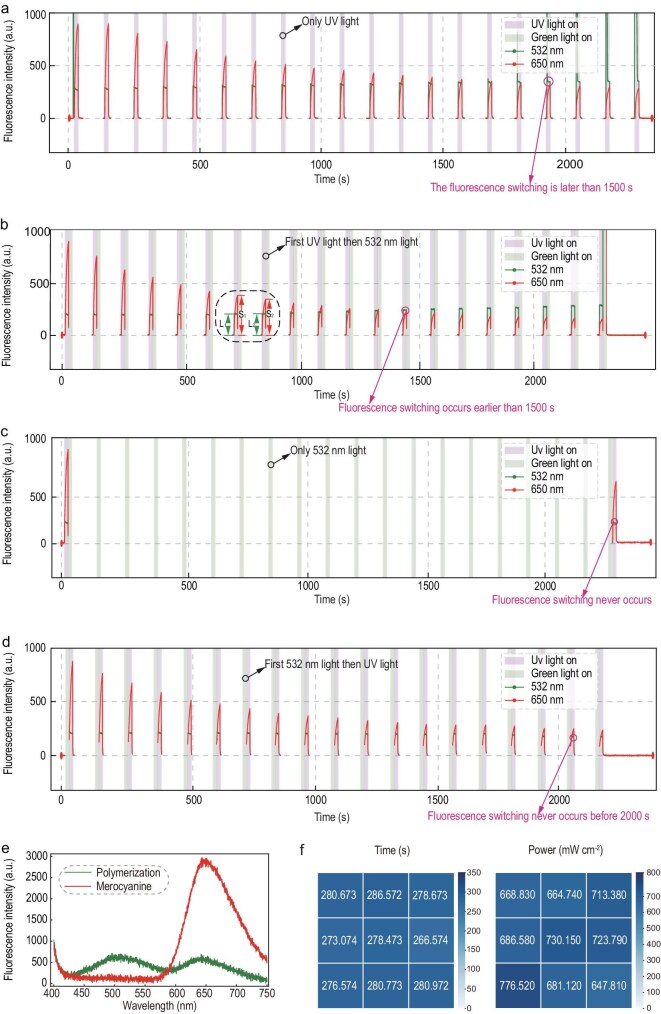
Fluorescence of the DCPI resin under UV and visible light irradiation. (a–d) Fluorescence intensity under four conditions corresponding to four cuvettes in Fig. 2a: (a) UV irradiation only, (b) UV irradiation followed by visible light, (c) visible light only, and (d) visible light followed by UV irradiation. (e) Fluorescence spectra of MC (red line) and polymer (green line). (f) Spatial uniformity of the fluorescence switching time and green fluorescence intensity of a square sample.

Figure 3a–d corresponds one-to-one with the four cuvettes shown in Fig. 2a, representing four scenarios: UV irradiation only, UV irradiation followed by visible light, visible light only, and visible light followed by UV irradiation. In the figures, the pink and green shaded areas indicate the time windows of UV and green light irradiation, respectively. The red and green lines represent the intensities of emitted red and green fluorescence, respectively. From the figures, it is evident that only in Fig. 3b does the fluorescence switching time occur before 1500 seconds, while the switching times for the other processes are significantly later than 1500 seconds. This gives us a sufficient time window for measurements. Additionally, it is noteworthy that the peak height of the red line in the figures reflects the MC content (S), while the height of the green step reflects the polymer content (L). Between the neighboring two tests, since MC and polymers are the reactant and product of the polymerization reaction, respectively, S_1_–S_2_ must be proportional to L_2_–L_1_. More discussions about the rationale behind the implementation of periodic alternating dual-color irradiation can be found in the first part of Supplementary Information.

Figure 3e presents the fluorescence spectra before and after fluorescence switching. Prior to fluorescence switching, red fluorescence was dominant over green fluorescence. After fluorescence switching, green fluorescence was dominant over red fluorescence, which reflects the transition from MC formation to polymer generation as the dominant process. Figure 3f shows the spatial uniformity of a square sample, which is a critical factor for ensuring reliable and reproducible results in downstream applications such as pattern recognition. The left panel depicts the fluorescence switching time at different positions while the right panel shows the green fluorescence intensity at these same positions. The different gradations of color are proportional to the numbers on them. High spatial uniformity can be observed for both the fluorescence switching time and green fluorescence intensity, which indicates consistent photochemical properties across the sample surface.

### Optical experiment

**Figure 4.**
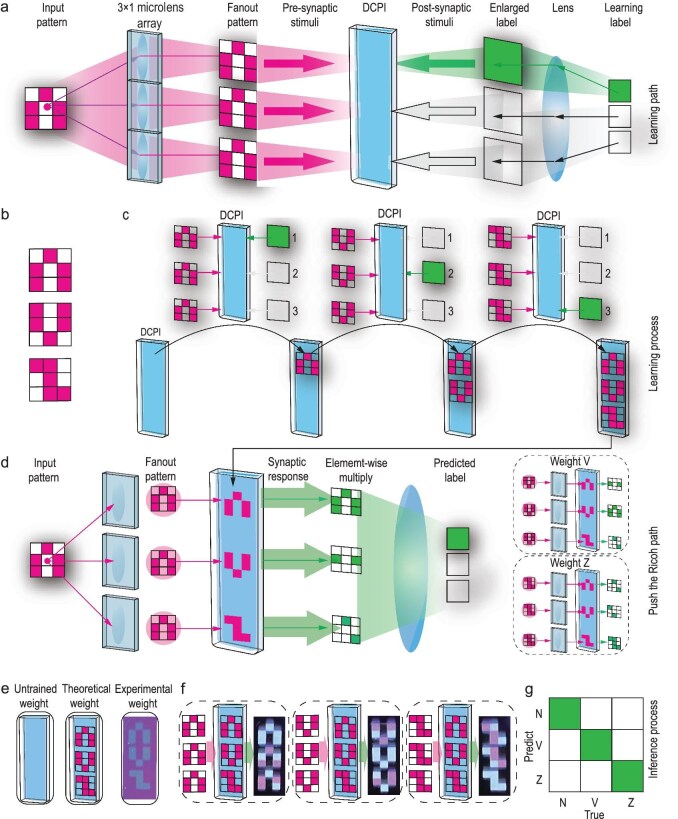
Application of the associative learning framework to pattern recognition in an optical experiment. (a) Optical pathway of the training process. (b) Patterns to be recognized. (c) Detailed training process for the three patterns. (d) Inference process. (e) Untrained weight (left), theoretical weight (middle), and experimentally obtained weight (right). (f) Recognition results for different input patterns: N (left), V (middle), and Z (right). (g) Comparison between the true value and prediction results.

Figure 4 illustrates the optical experiment used to apply the established associative learning mechanism to pattern recognition. The training process involved illuminating a pattern with UV irradiation on the left side and projecting the recognition result using visible light on the right side. This resulted in the formation of a polymer pattern on the SP film in the middle, which functioned as the weight. The regions of the SP film irradiated sequentially by UV and visible light underwent associative learning and emitted a green fluorescence under subsequent UV irradiation. In contrast, regions not exposed to both irradiation types only emitted red fluorescence under subsequent UV irradiation. The inference process involves projecting a pattern via UV irradiation onto the SP film, where the interaction with pretrained weights generates a green fluorescence pattern​ that serves as the recognition result. The intensity and distribution of the green fluorescence was then used to identify the input pattern. In the optical experiment, the ‘N,’ ‘V,’ and ‘Z’ patterns were used as illustrative examples.

Figure 4a shows the optical pathway of the training process. First, the ‘N’ pattern was fanned out three times and projected onto the initial SP film by UV irradiation. The number of fan-outs corresponded to the type of patterns to be recognized to ensure that all potential recognition outcomes were accounted for during the training process. Subsequently, visible light was projected onto the SP film to initiate the associative learning process, where polymerization was activated in regions exposed to both UV and visible light. The above procedure was repeated for the ‘V’ and ‘Z’ patterns to complete the training process for all three patterns, as illustrated in Fig. 4b and c. Thus, the SP film was effectively trained to recognize each input pattern by the establishment of specific polymerization patterns that corresponded to their respective fluorescence outputs. This process mimics the associative learning mechanism of biological memory in which stimuli are linked to responses through repeated exposure and interaction.

Figure 4d shows the inference process. The input UV pattern was also subjected to the fan-out process before being passed through the trained weight. Note that, while the schematic diagram divides the patterns into blocks with and without green fluorescence, every block in each pattern emitted green fluorescence in the experiment. The strongest green fluorescence intensity corresponded to blocks where the UV input pattern and weight pattern overlapped fully while other blocks emitted less green fluorescence owing to partial overlap. The intensity of the green fluorescence was used to infer the correct input pattern.

Figure 4e compares the untrained weight, theoretical weight, and experimentally obtained weight from left to right. Figure 4f presents the experimentally obtained weights and inference results after inputting the UV patterns of ‘N,’ ‘V,’ and ‘Z.’ The distribution of the green fluorescence intensity on the film matched expectations, which confirmed the accuracy of the inference process as shown in Fig. 4g. The clear large figures of the experimentally obtained weights and inference results are shown in Figs. S11–S14. The non-idealities of the experiment, which are mainly due to diffusion of liquid, can be observed more clearly.

### Simulation

**Figure 5.**
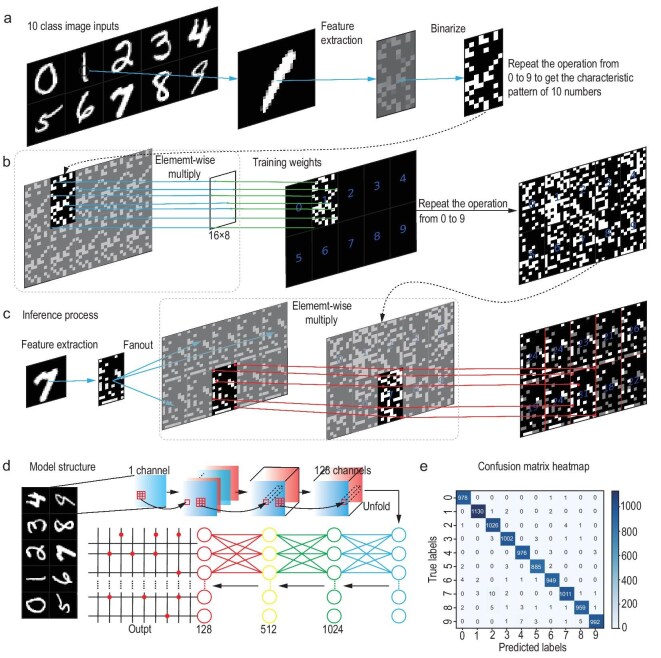
Simulation applying the established associative learning framework to handwritten digit recognition. (a) Simplification of handwritten digits pattern to 16 × 8 binary matrices. (b) Training process. (c) Inference process. (d) Model structure. (e) Performance evaluation.

To evaluate the potential scalability and broader applicability of the associative learning framework, a simulation experiment was conducted where it was applied to the recognition of handwritten digits. We employed a traditional convolutional neural network (CNN) to preprocess the handwritten digit patterns. Features extracted by the CNN were stored in a 16 × 8 matrix for each digit. After binarization, the matrix elements were reduced to binary values (0 or 1) to effectively represent the handwritten digits in low-resolution and black-and-white form, as illustrated in Fig. 5a. This preprocessing step reduced the complexity of the input and made it conceptually analogous to the letter patterns used in the optical experiment. The feature matrices were extracted for digits 0–9. The associative learning framework was then applied to the preprocessed patterns. For the training process, each binary feature matrix representing a digit was multiplied elementwise with a 16 × 8 matrix consisting entirely of ‘1’ elements, which was equivalent to the process of projecting visible light onto the UV-illuminated patterns in the optical experiment. The training also required a fan-out operation where each pattern was replicated 5 × 2 = 10 times to associate each digit with ten possible recognition outcomes. This process was repeated for all ten digits to result in a trained weight matrix with dimensions of 16 × 8 × 5 × 2 elements where each element was either 0 or 1, as shown in Fig. 5b, that encoded the relationship between the input patterns and their corresponding recognition results. For the inference process, the feature matrix of an input digit was similarly subjected to a fan-out operation into a 5 × 2 form. Each replicated matrix was then multiplied elementwise with the trained weights. Among the resulting 5 × 2 matrices, the matrix corresponding to the correct digit contained the largest number of ‘1’ elements, which was analogous to the blocks with the strongest green fluorescence intensity in the optical experiment. For instance, if the input digit was ‘7,’ the associated weight pattern would align most closely with the input pattern and result in the highest count of ‘1’ elements. This logical process is visualized in Fig. 5c, and the complete model structure is summarized in Fig. 5d. Figure 5e shows the confusion matrix of the simulation results, which demonstrates the reliability of the developed framework for handwritten digit recognition (see Fig. S16 for the corresponding training loss). The results aligned closely with expectations, which confirmed that the framework can be effectively scaled up from recognizing simple patterns to more complex tasks.

## DISCUSSION

The above associative learning framework provides a simple yet complete demonstration of an optical training and recognition algorithm that corresponds directly to the theory of synaptic plasticity with the steps of the process corresponding to presynaptic stimuli, postsynaptic stimuli, and synaptic responses (Fig. 4a and d). The underlying mechanism is fundamentally a form of element-wise multiplication (Fig. S15 [35]) or matrix-vector multiplication (MVM). While MVM is a simple mathematical operation, it serves as the foundational building block for the convolutional neural network, which achieves high performance through the combination of numerous MVMs and nonlinear activation functions in applications such as image recognition. Beyond MVM, our method incorporates the fan-out operation, which substantially enhances the model’s ability to solve complex problems and improves its robustness. The patterning of resin is the process that physically forms the network’s weights and it directly determines the accuracy of experimental results. It lays a foundation for training ONNs in a manner analogous to the learning process of humans. It is worth noting that not all letters can be distinguished by our device. For example, ‘Q’ and ‘O’ are inapplicable. Because the pattern of ‘Q’ can cover the pattern of ‘O’ totally. When we input the ‘O’ pattern, we will have the same green light intensity at two different output sites.

**Figure 6.**
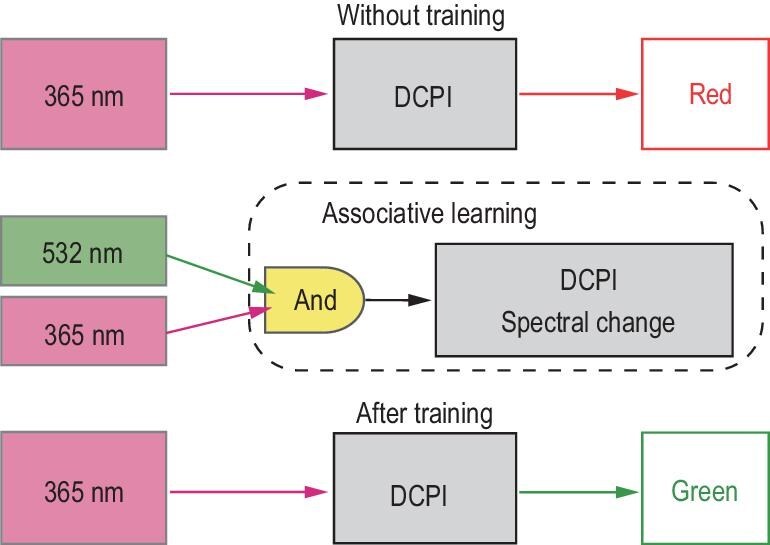
Logical flow of the associative learning framework.

Figure 6 summarizes the logical structure of the associative learning framework. UV irradiation of the DCPI resin causes SP to transform into MC, which emits a red fluorescence under UV irradiation. In contrast, visible light irradiation of the DCPI resin causes no chemical interaction. However, when UV irradiation is projected first followed by visible light, associative learning is activated, and the polymerized product emits green fluorescence when subsequently irradiated by UV light.

**Figure 7.**
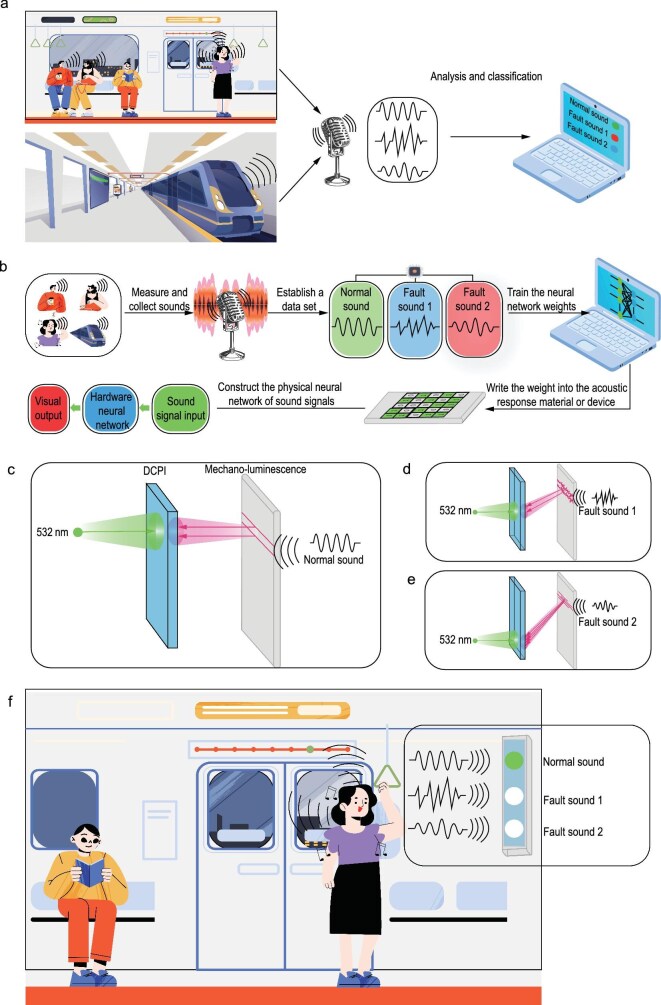
Comparison between three different methods for sound recognition. (a) Electronic computer. (b) Bottom-up fabrication method of hardware neural network. (c–f) Top-down fabrication method of optical neural network based on associative learning.

To clarify the difference between the electronic computer, mainstream approach to fabricate hardware neural networks and the associative learning approach, consider the following application scenario: during metro operation, diverse sounds are generated—some are normal operational noises, while others indicate mechanical malfunctions. The goal is to develop a physical device that analyzes these sounds to identify potential faults in the metro system. The electronic computing approach employs a microphone to capture acoustic signals, converts mechanical vibrations into electrical signals, transmits these signals to the electronic computer, where specific algorithms classify them, with results displayed on a monitor (Fig. 7a). The mainstream approach for fabricating hardware neural networks first requires measuring various sound signals representing normal and faulty conditions to establish a dataset. Based on this dataset, neural networks are trained on electronic computers to obtain weights. The response characteristics of specific sound-responsive materials or devices are then used to represent these weights. Finally, arranging these materials/devices into network architectures yields a physical neural network capable of sound signal reception and classification. As this fabrication method constructs the system by stacking fundamental building blocks into an integrated whole, it is termed ‘bottom-up’ (Fig. 7b). For the associative learning approach proposed in this work, the process begins with a layer of mechano-luminescence material that converts mechanical vibration signals (sound) into optical patterns. Each sound type corresponds to a specific light pattern, which is analogous to the letter patterns shown in Fig. 4. Suppose that the wavelength of the light pattern is tuned to be UV by carefully choosing the mechano-luminescence material, then we should project visible light onto a certain region of DCPI simultaneously when the sound comes. This training procedure is repeated for all sound types, with each unique sound pattern being mapped to distinct illumination positions on the DCPI surface (Fig. 7c, d, and e). Notably, this training mechanism shares conceptual similarity with software-based neural network training. During inference, both bottom-up and top-down neural networks are fundamentally identical in operational principles. For this application scenario, fault detection is achieved through brightness analysis: specific regions of the DCPI can be labeled as ‘fault’ indicators (Fig. 7f). When these annotated regions exhibit brighter fluorescence compared to others, it signifies metro system anomalies. Further classification about fault is achievable by correlating distinct regions with specific fault categories.

This example demonstrates that conventional electronic computing solutions require expensive, complex instrumentation to address such problems. While their mature development ensures superior performance, optical neural networks exhibit distinct advantages. These include no external power requirements during operation, exceptional robustness (for example, more possibly maintaining functionality after collisions compared with the electronic system due to its edge computing nature), and the capability for extremely low-cost large-scale deployment. On the other hand, though idealized, this demonstration reveals core features of associative learning-based fabrication when compared with the mainstream bottom-up approach: (1) weight-determination integration: unlike conventional methods requiring separate software/hardware processes, associative learning achieves simultaneous weight determination and physical implementation through pure physical training. For our DCPI approach, co-illumination of UV and visible light at designated positions completes neural network training. (2) Top-down self-organization: macroscopic sound/light inputs automatically configure microscopic material responses without requiring knowledge of specific photochemical reaction sites or pattern formation mechanisms. The system autonomously determines necessary neuron connections based on input-output mapping requirements, justifying its designation as ‘self-learning’ or ‘the nature knows.’

To our knowledge, there have been two prior works on implementing associative learning using optics [28,29]. In Hang Zhang’s work, the two stimuli for associative learning were light illumination and thermal effects, with the physical effect after learning being the melting of the gel. This represents the earliest known implementation of optical associative learning and features the advantage of ‘forgetting capability.’ In James Y. S. Tan’s work, the two stimuli were optical pulses with fixed phase differences, resulting in phase change material (PCM) amorphization and increased transmittance after learning. This approach achieved all-optical associative learning and demonstrated advantages in parallel processing of different wavelength light signals. Compared with these predecessors, our work demonstrates two principal advantages: (1) following biological Pavlovian conditioning principles where stimulus sequence is critical (meat must precede bell sound), our optical analogy requires strict temporal sequence of UV illumination followed by visible light exposure, achieving closer biological fidelity. (2) While Hang Zhang’s method relies on thermal effects that are challenging to precisely control, and James Y. S. Tan’s approach requires fixed phase differences demanding high device precision, our DCPI-based method offers enhanced implementability for associative learning. This implementability brings the potential to fabricate an optical neural network with the ‘top-down’ method.

Regarding future research directions, two critical challenges must be addressed. First, the second polymerization reaction in DCPI is inherently irreversible, which precludes active forgetting—a limitation mirroring biological memory systems where humans can consciously acquire knowledge but lack mechanisms to erase specific memories. While reversible reaction materials (e.g. Hang Zhang *et al.*’s work [28]) might theoretically resolve this issue, such materials typically compromise synaptic weight stability, making long-term memory retention (e.g. decades-long human-like memory storage) challenging. This fundamental trade-off between reversibility and material stability suggests that irreversible reactions may remain necessary for practical memory applications. So, in-depth investigation of material photochemical reaction mechanisms and development of scenario-specific materials represent crucial future research directions. Second, we can only solve very elementary classification problems. That’s because we only realized the linear component of the neural network (element-wise multiplication between matrixes) and we have only one layer. If we hope to solve more complex problems, we need the non-linear part and more layers. How to realize the non-linear part as well as how to combine the linear part and nonlinear part together based on associative learning is still an open question.

## METHODS

### Optical experiment

The DCPI resin was synthesized by mixing 19.0 g of pentaerythritol tetraacrylate (PETA) with 1.0 g (5 wt%) of triethanolamine solution containing 2.4 mg (0.01 wt%) DCPI. To measure the fluorescence, a coaxial optical path was built by installing a 19-mm-wide dovetail guide rail (Jingcui Optics) with a length of 600 mm on the optical platform and using the corresponding guide rail slider as a fixed base. The UV irradiation source (UV-LED point light source) irradiated along the guide rail direction. The UV irradiation passed through a planoconvex lens with a focal length of 50 mm (material: N-BK7, D = 25.4 mm) and another planoconvex lens with a focal length of 25 mm (material: N-BK7, D = 25.4 mm) to converge at a spot with a diameter of 1 mm. The two lenses were installed into a lens sleeve (Jingcui Optics, internal thread) and fixed by an external thread snap ring. The two lenses were both installed on the same 30-mm-wide coaxial mounting plate with a thickness of 12.7 mm (Jingcui Optics). The 1-mm-diameter irradiation spot was used to illuminate a 10 mm × 1 mm quartz cuvette filled with DCPI resin and fixed by the fixed column lens mounting seat. With this setup, the UV irradiation source, lens group, and cuvette with DCPI resin were all on the same coaxial optical path. Fluorescence was detected by using an Ocean Optics spectrometer (QEPRO). The spectrometer probe was placed on the side of the cuvette closer to the UV irradiation source and aligned with the area of the DCPI resin excited by the irradiation spot. The reflected light mode was selected, and the software provided by the Ocean Optics spectrometer was used to eliminate background light. The UV irradiation source was turned on, and data were acquired for 300 seconds. The corresponding schematic diagram and experimental photograph are shown in Fig. S1 to Fig. S3.

The simulation method is in the Supplementary Data.

## Supplementary Material

nwag029_Supplemental_File

## Data Availability

The data and code that support the findings of this study are available from the corresponding author upon reasonable request.
