# Supplementary material for: Pavlov’s experiment-inspired optical neural networks based on dual-color fluorescence switching effect
Source: Natl Sci Rev. 2026 Jan 19;13(5):nwag029. doi: 10.1093/nsr/nwag029 (PMC12951521; doi:10.1093/nsr/nwag029)
Supplement: nwag029_Supplemental_File [file nwag029_supplemental_file.pdf]

Pavlov's experiment-inspired optical neural networks based on dual-color fluorescence switching effect

The rationale behind the implementation of periodic alternating dual-color irradiation

The periodic visible light and UV irradiation was critical for ensuring the precision and reliability of the associative learning process. During visible light irradiation, it was essential to avoid signal overlap for fluorescence detection. The laser used to induce polymerization was turned off during this step to prevent interference between the incident green light and emitted green fluorescence and ensure a clear separation of the input and output signals to maintain the integrity of the experimental results. The periodic UV irradiation was crucial to preserve the sequence of stimuli, which was a key principle of Pavlov's experiment. There, an associative response was established by always presenting the bell before the food. Analogously, the UV irradiation (i.e., stimulus to generate MC) had to precede the visible light irradiation (i.e., stimulus for polymerization) to trigger the underlying chemical mechanism, where SP must be converted to MC first before MC can be polymerized. Reversing the order by projecting visible light before UV irradiation would disrupt the reaction and hinder the learning process.

The periodic UV irradiation also helped minimize secondary reactions23. As noted earlier, while UV irradiation primarily converts SP to MC, it can also weakly induce the polymerization of MC. Periodic UV irradiation reduced the occurrence of this minor secondary reaction by limiting the exposure time without compromising the fluorescence intensity, which is directly proportional to the UV irradiation intensity. Based on the fluorescence intensity profile in Figure 3a, the UV irradiation duration was set to 10 s, which corresponded to the maximum red fluorescence intensity and indicated when the MC generation peaked before it started to be consumed by the secondary polymerization. This effectively enhanced MC production and reduced unwanted side effects to enable precise control of the photochemical reaction dynamics.

Simulation method

The overall approach involves preprocessing images with a CNN to extract fundamental features, followed by associative learning-based classification of the processed data. This methodology not only significantly reduces input data complexity but also ensures visual and structural consistency with the letter patterns used in our optical experiments, establishing a foundation for cross-modal transferability. About the dataset, we employed the MNIST handwritten digit dataset, containing 10 classes of grayscale images with 28×28 pixels per single-channel input.

CNN Preprocessing: The model takes 28×28 two-dimensional grayscale images as input. The first layer is a convolutional layer using 32 3×3 convolutional kernels for feature extraction. To maintain spatial dimensions, appropriate padding was applied, producing 32-channel 28×28 feature maps. A ReLU activation function follows the convolution to enable nonlinear transformation. The subsequent two convolutional layers expand the channel count to 64 and 128, respectively, still using 3×3 kernels and ReLU activations. The final output comprises 128 28×28 feature maps, forming a 128×28×28 tensor that is flattened into a vector for input to fully connected layers. The flattened vector passes through three fully connected layers sequentially: the first outputs a 1024-dimensional vector, the second compresses it to 512 dimensions, and the third generates a 128-length image representation vector. All layers use ReLU activation. This 128-dimensional feature vector is then fed into the "material layer," where similarity computation with embedding vectors representing 10 categories enables classification prediction. Model training employs a categorical cross-entropy loss function, with prediction accuracy used for performance evaluation.

Transition from CNN to Associative Learning: To ensure CNN-extracted features align with the material layer's processing mechanism, we constructed a simulated material layer before associative learning training. This layer replaces the original material layer's binarization mechanism with a smooth function to enable differentiability, facilitating end-to-end training via backpropagation. This modification allows joint optimization of the CNN and simulated material layer, extracting features suitable for associative learning.

Classification via Associative Learning: During training, we simulated the "exposure" process from optical experiments. Specifically, each binarized feature matrix undergoes element-wise multiplication with a 16×8 matrix filled with ones, analogous to uniformly illuminating the digit pattern to establish semantic correspondence between input patterns and output responses. To support classification, a fan-out mechanism was introduced: each input pattern is replicated 5×2=10 times and paired with category-specific encodings. This process creates multiple connections between each digit and classification labels. The final training phase generates a 16×8×5×2 four-dimensional weight matrix with binary elements (0/1) representing association strength between input patterns and class labels (see Figure 5b). During inference, the 128-dimensional feature matrix from CNN processing

undergoes 5×2 fan-out. Each submatrix undergoes element-wise multiplication with the trained weight matrix, producing a 5×2 matrix where larger values indicate higher matching scores between specific positions (class labels) and input patterns. The system counts "1"s in each position to determine the most matched output label. This mimics optical experiments where the green fluorescence intensity region indicates recognition activation. For example, input "7" aligns strongly with its corresponding weight matrix, yielding the highest "1" count at its label position (see Figure 5c). The complete model architecture is shown in Figure 5d.

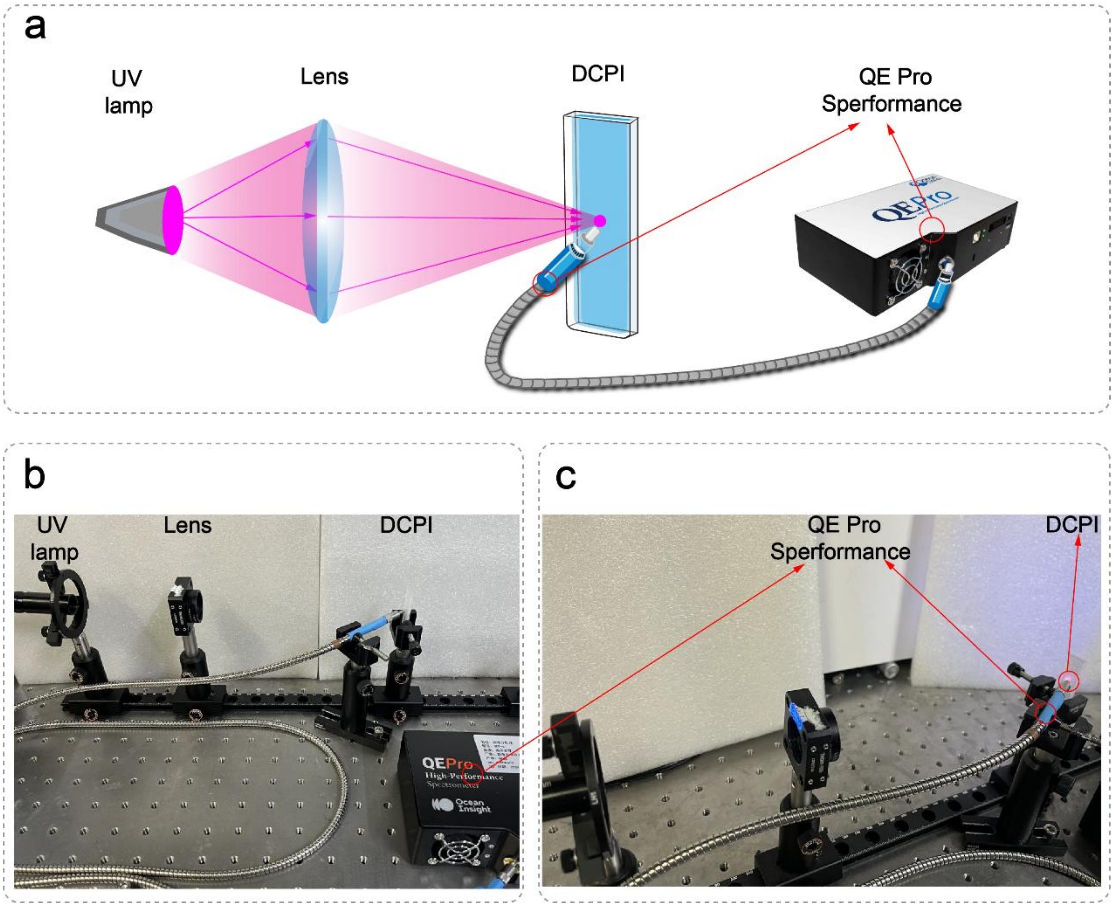

Figure S1. The schematic diagram (top panel) and its experimental photograph (bottom panel) of fluorescence property measurement under UV irradiation. **Figure b** and **Figure c** are photographs of the same set of instruments taken from different angles.

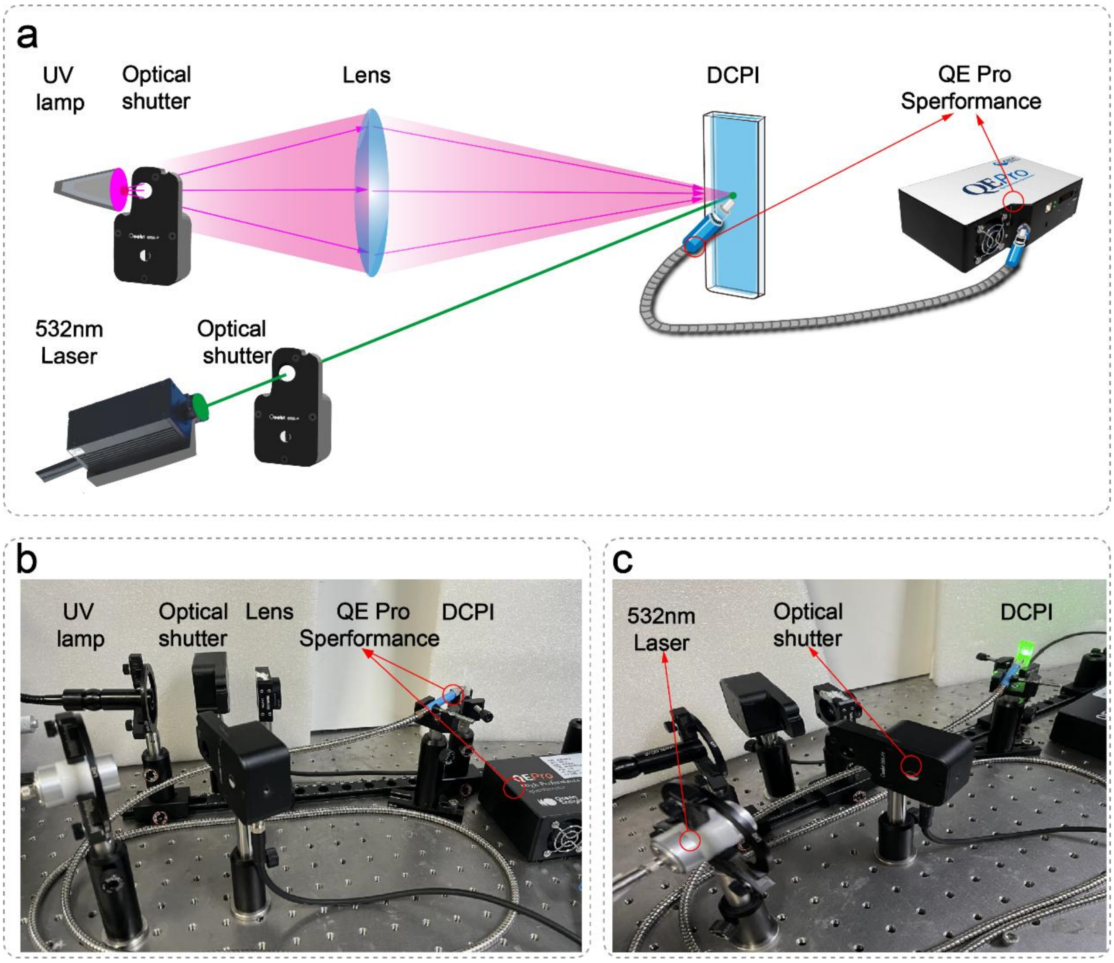

Figure S2. The schematic diagram (top panel) and its experimental photograph (bottom panel) of fluorescence property measurement under UV and visible light (532 nm) irradiation simultaneously. **Figure b** and **Figure c** are photographs of the same set of instruments taken from different angles.

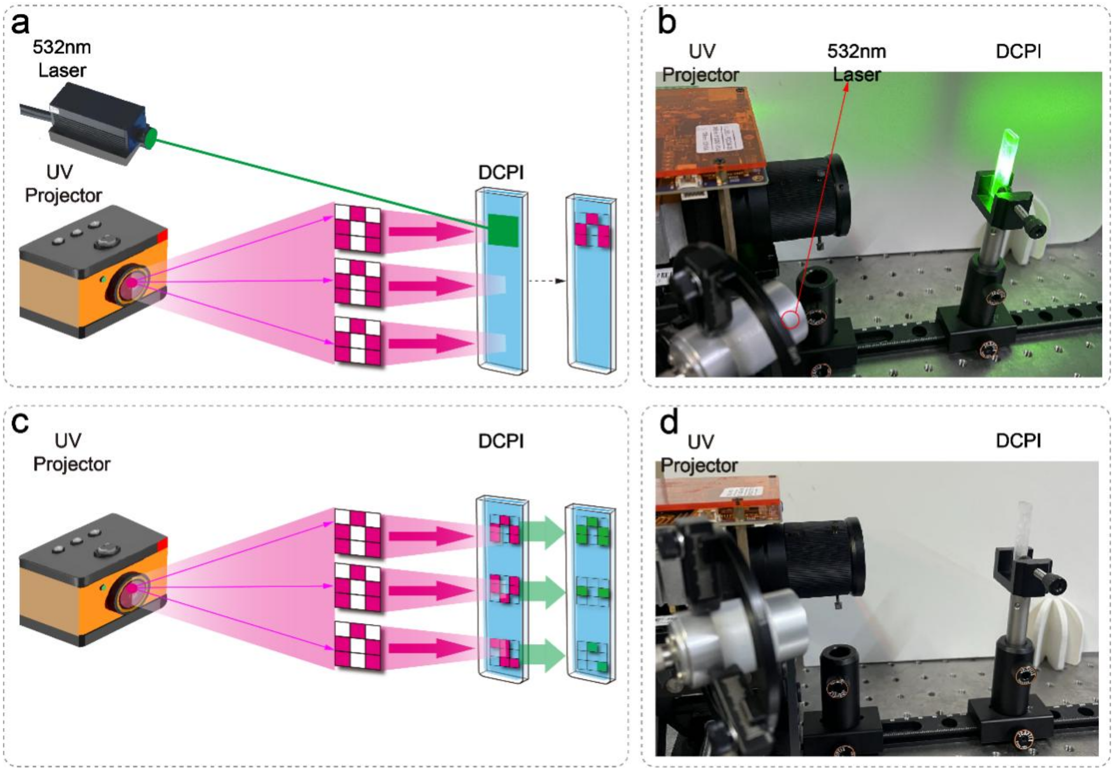

Figure S3. The schematic diagram and its experimental photograph of training and inference process. Figure a and Figure b are the training process while Figure c and Figure d are the inference process.

Figure S4, S5 and S6 show ^{1}H, ^{13}C and ^{19}F NMR spectra of **DCPI** synthesized in our experiment. They are consistent with the reference.

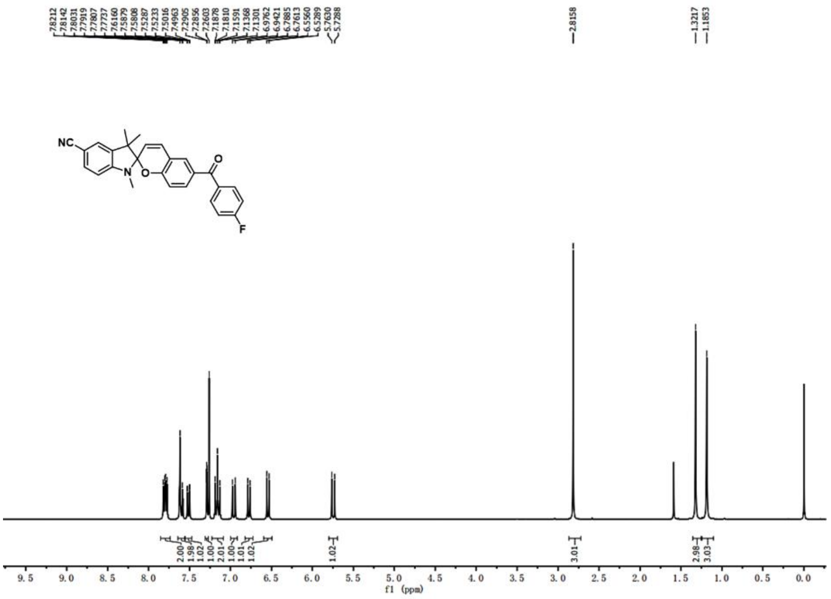

Figure S4. ^{1}H NMR (500 MHz, $CDCl_3$): δ (ppm) = 7.82-7.77 (m, 2H), 7.61-7.58 (m, 2H), 7.51 (dd, J = 13.5, 2.7 Hz, 1H), 7.18 (d, J = 3.4 Hz, 1H), 7.14 (t, J = 11.1 Hz, 2H), 6.96 (d, J = 17.0 Hz, 1H), 6.77 (d, J = 13.6 Hz, 1H), 6.54 (d, J = 13.5 Hz, 1H), 5.74 (d, J = 17.1 Hz, 1H), 2.81 (s, 3H), 1.32 (s, 3H), 1.18 (s, 3H).

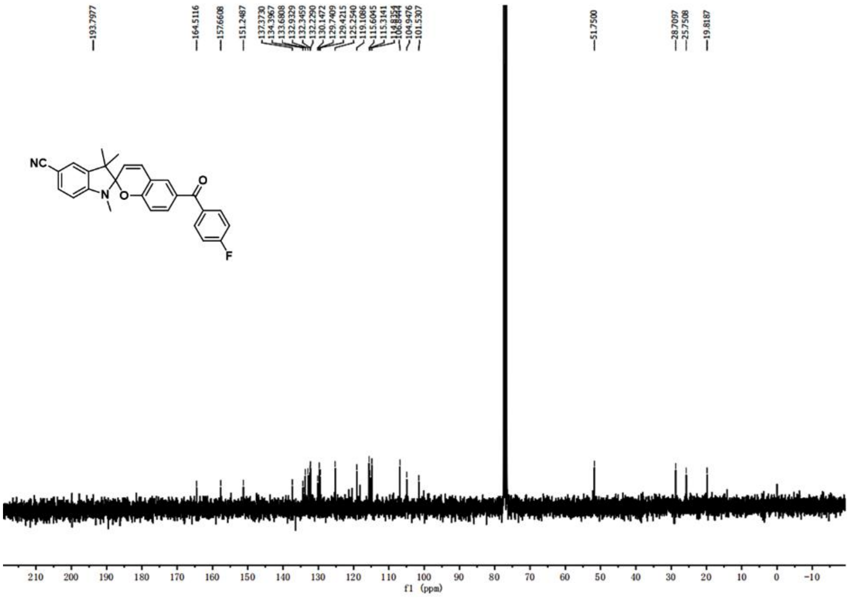

Figure S5. ^{13}C NMR (125 MHz, $CDCl_3$): δ (ppm) = 193.79, 166.13, 164.51, 157.66, 151.24, 137.37, 134.39, 133.68, 132.93, 132.34, 132.22, 130.14, 129.74, 129.42, 125.25, 119.22, 119.10, 115.60, 115.31, 114.83, 106.84, 104.94, 101.53, 51.75, 28.70, 25.75, 19.81.

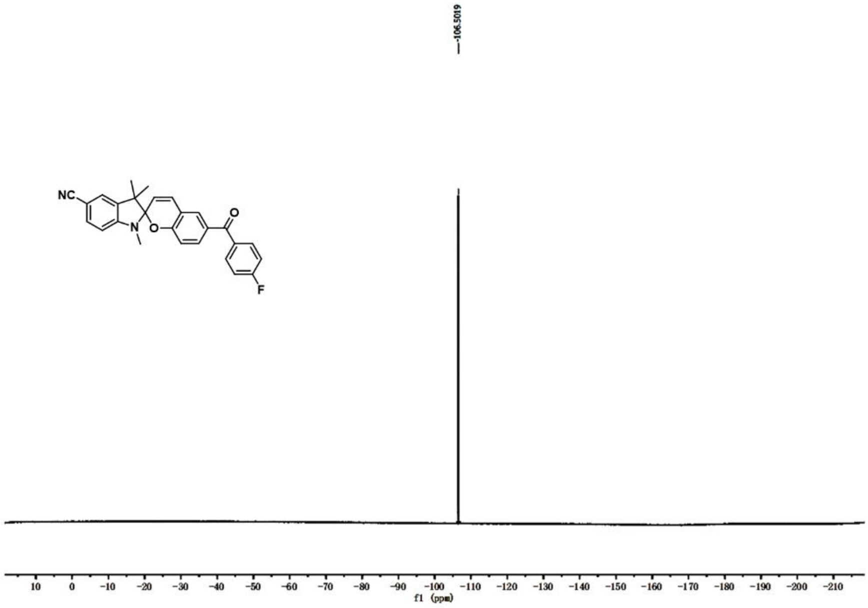

Figure S6. ^{19}F NMR (471 MHz, $CDCl_3$): δ (ppm) = -106.50. HRMS (+ESI) m/z calcd. for $C_{27}H_{22}FN_2O_2$ $(M+H)^+$: 425.166 found 425.174.

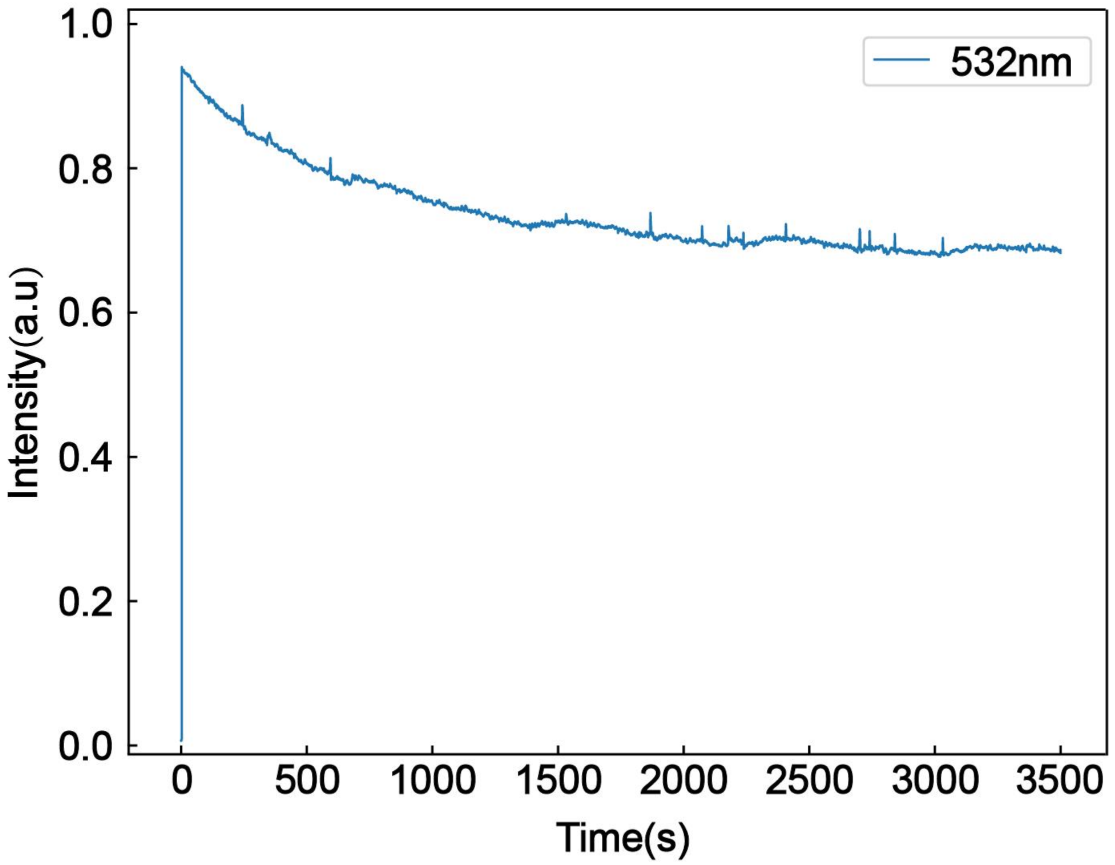

Figure S7. We project a UV light continuously on DCPI after polymerization and then measure the fluorescence intensity changing with time. The power of UV light is 15 mW/cm^2. The fluorescence intensity exhibited an initial sharp decline followed by gradual stabilization.

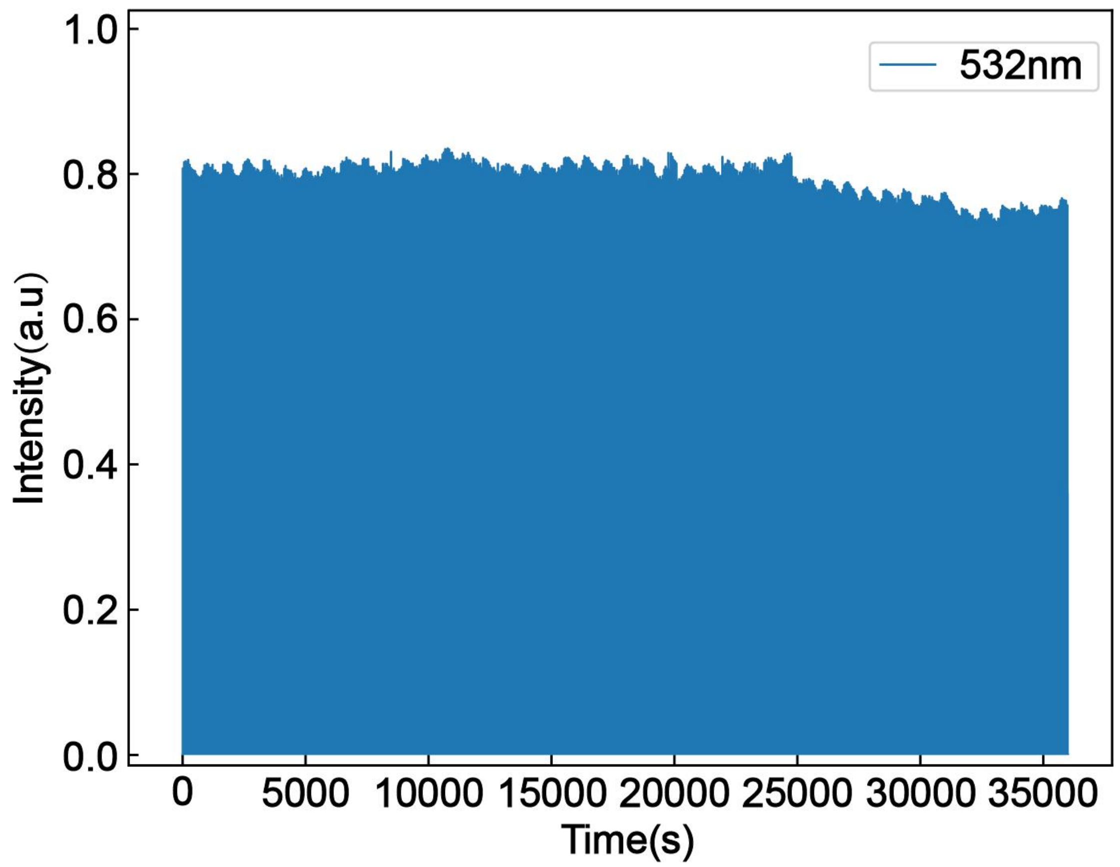

Figure S8. We project a periodic UV light on DCPI after polymerization and then measure the fluorescence intensity changing with time. The UV light is on for 5 s and off for 50 s. The power of UV light is 15 mW/cm^2. The fluorescence intensity decay rate exhibited in this experiment was slower compared to that observed in **Figure S7**.

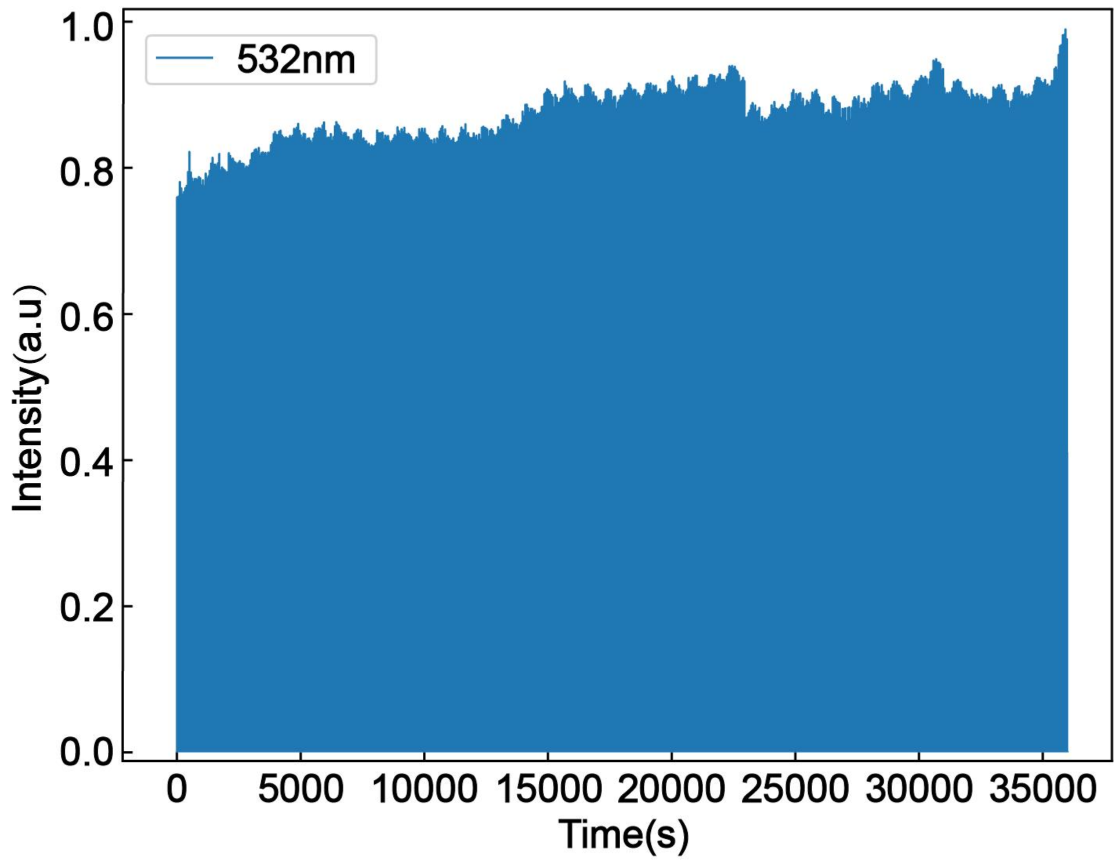

Figure S9. We project UV and visible light (532 nm) alternately on DCPI after polymerization and then measure the fluorescence intensity changing with time. The UV light is on for 5 s and off for 50 s. The visible light is on for 50 s and off for 5 s. The intensity of UV light and visible light is 15 mW/cm^2 and 135 mW/cm^2, respectively. Notably, the fluorescence intensity exhibited an upward trend instead of the anticipated decay. The visible light appears to exert a restorative effect on the fluorescence performance of the material. Although this phenomenon appears peripheral to the core focus of this study, it may worth further investigation.

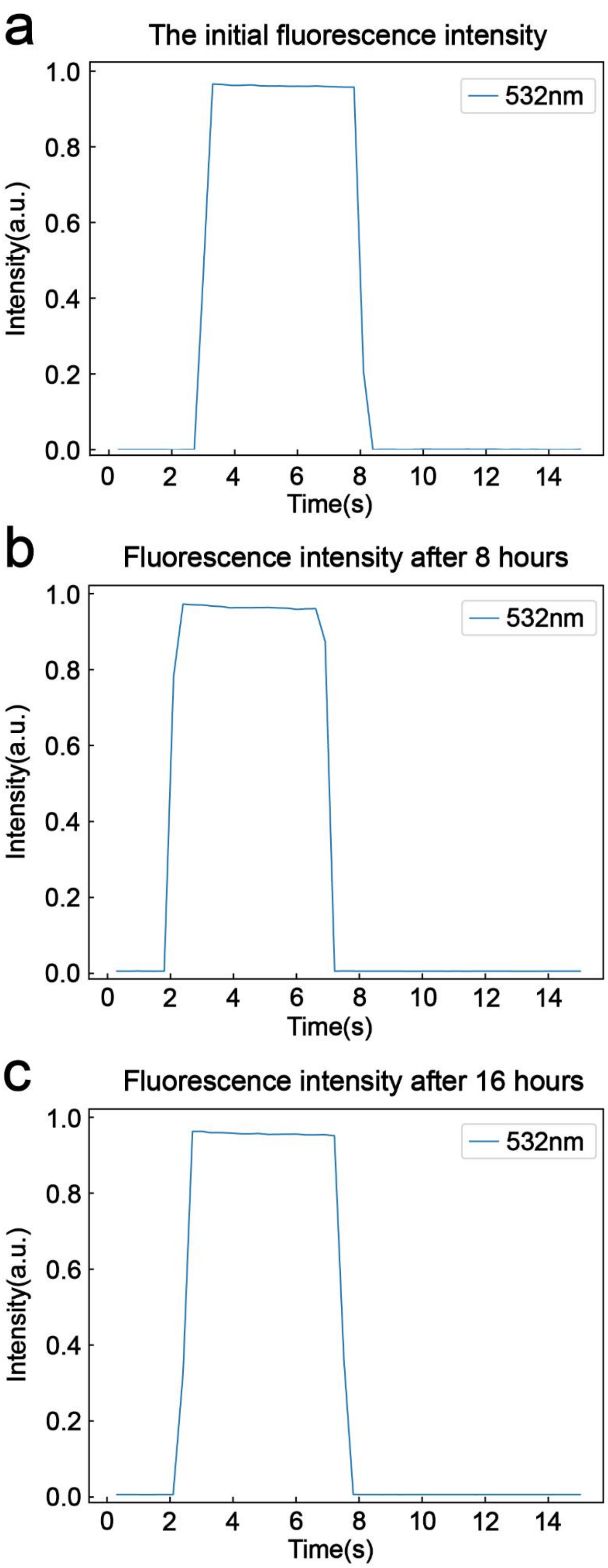

Figure S10. The fluorescence intensity variation of DCPI polymer after 8 and 16 hours without external stimuli.

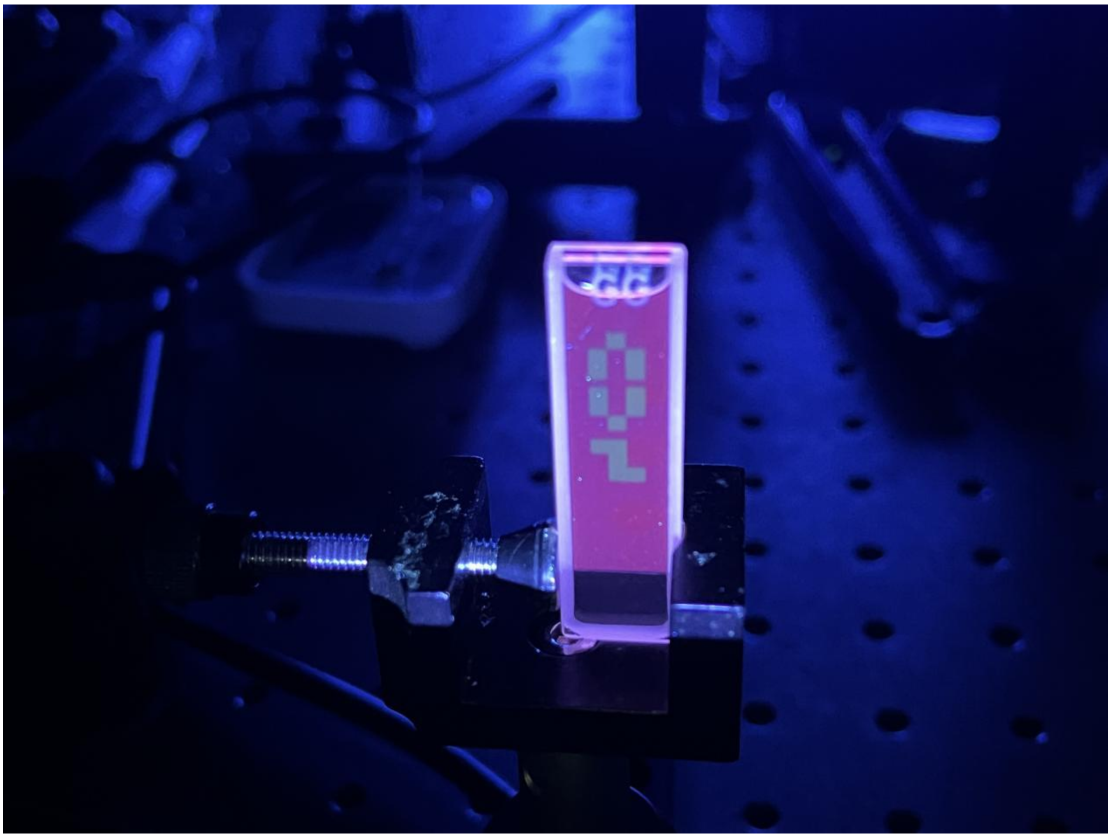

Figure S11. The photograph of weight obtained in experiment.

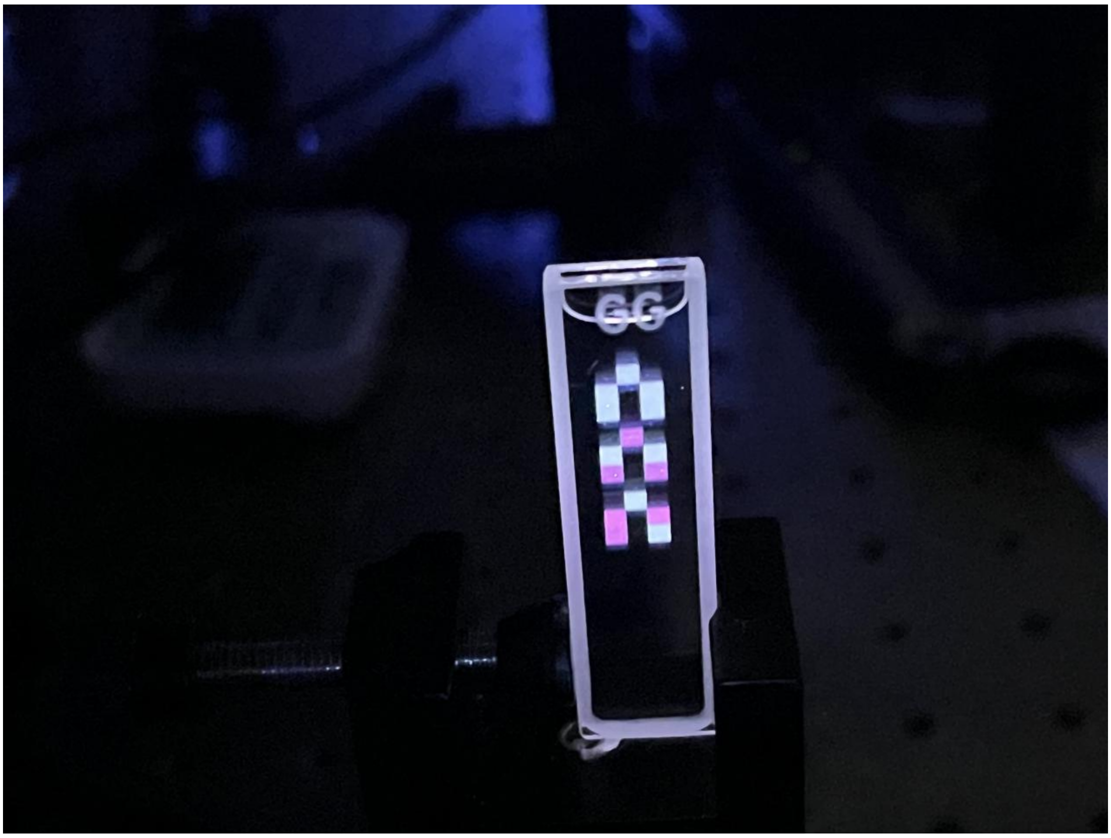

Figure S12. The photograph of “N” result obtained in experiment.

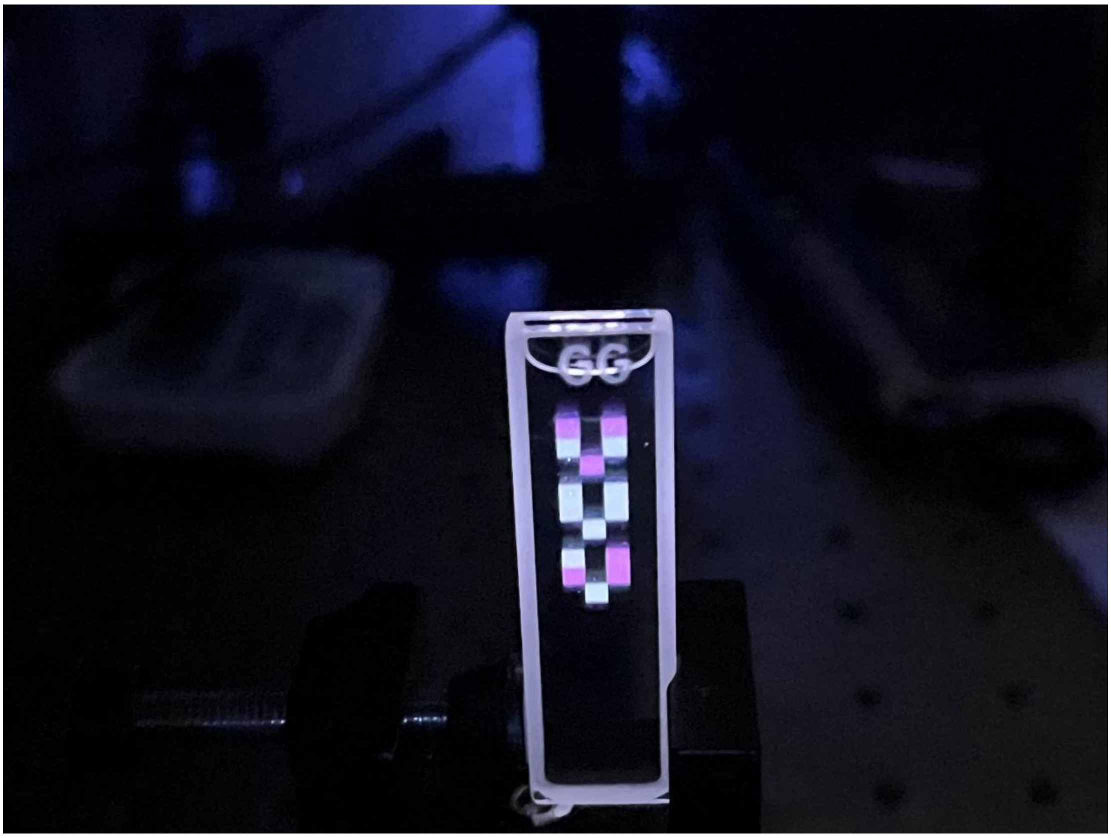

Figure S13. The photograph of “V” result obtained in experiment.

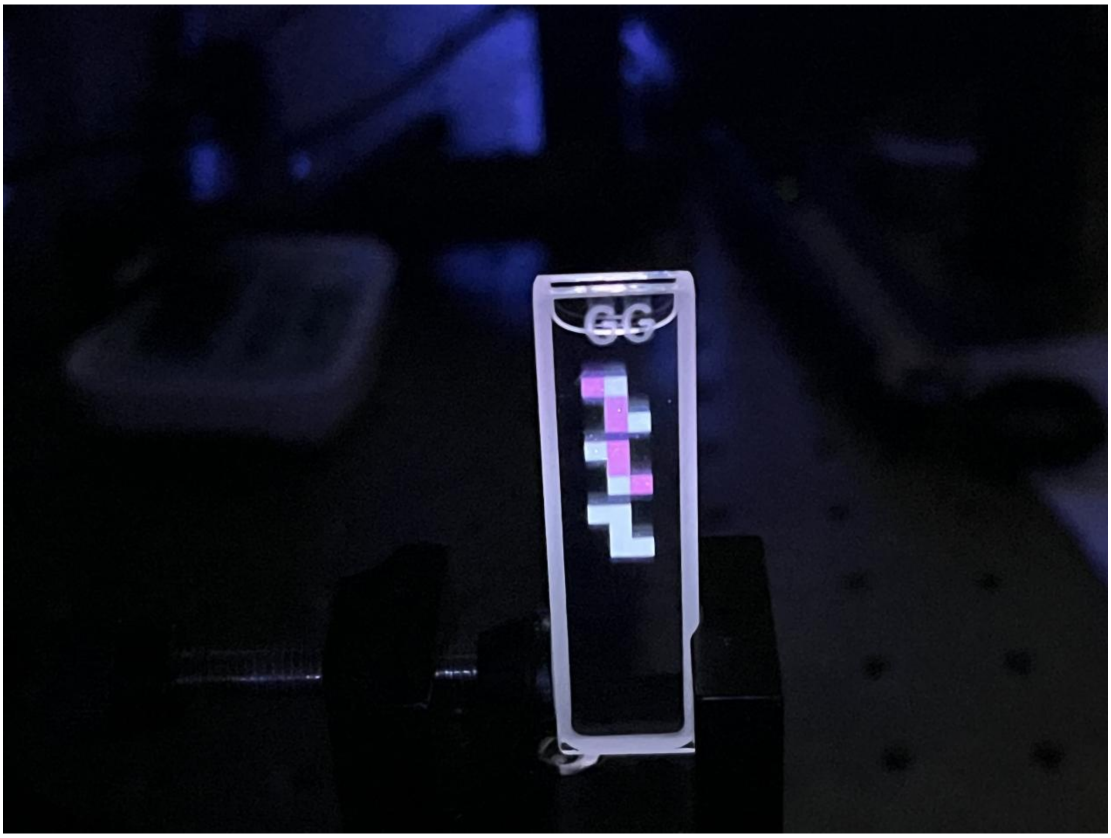

Figure S14. The photograph of “Z” result obtained in experiment.

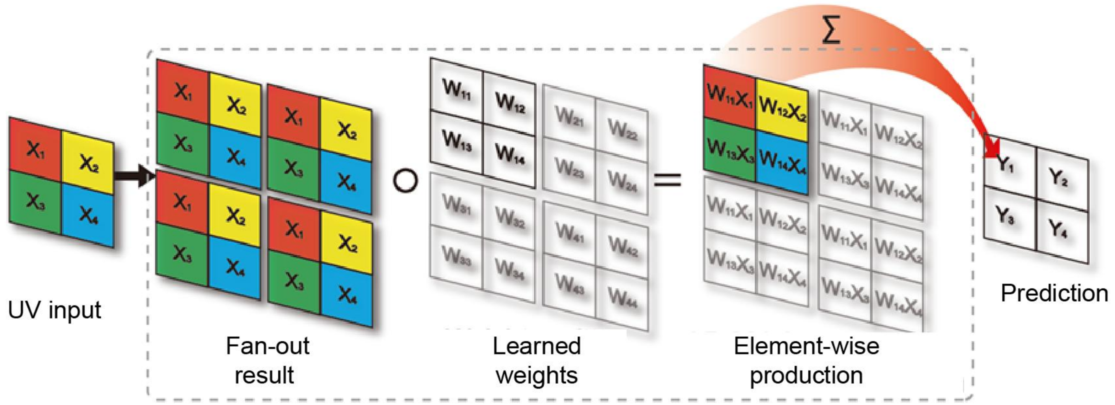

Figure S15. The schematic diagram of element-wise multiplication.

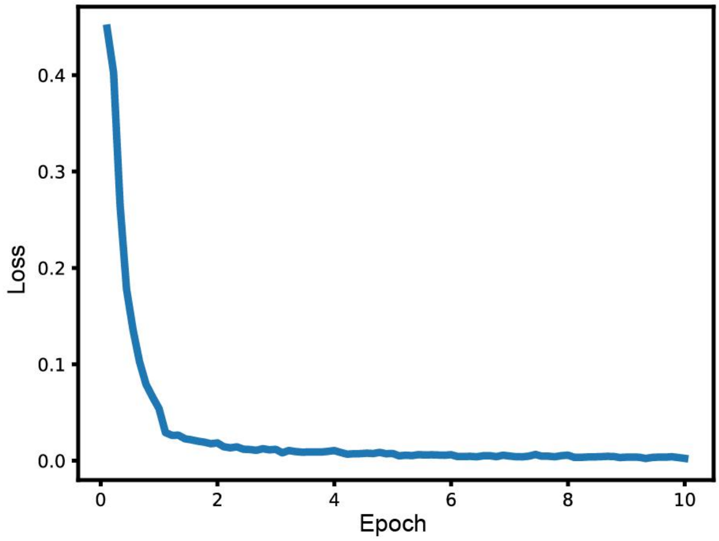

Figure S16. Training loss for MNIST classification

Table S1. Comparison between our work and two former works

Different works	Tan et al., 2022	Zhang et al., 2019	Our work
**Core Materials**	Integrated photonic platform + phase change material	Agarose hydrogel + lipoic acid	spiropyran (SP)/ merocyanine(MC)
**Associative Stimulus Type**	A specific optical phase difference Δφ	Combination of dual stimuli: light+ thermal	Two - wavelength light stimulation
**Core Learning Mechanism**	The crystalline - amorphous phase transition	Light - induced pH change and heat-induced hydrogel melting	Photo-induced chemical conversion + irreversible polymerization
**Memory Characteristics**	Non - volatile	Volatile	Non - volatile
**Key Advantages**	All - optical architecture	Forgetting and memory recovery.	Correct time sequence; Low manufacturing costs; Easy to perform.

Table S2 Comparison between different DCPI materials

Material	On/off Ratio	Reversibility	Stability	Energy Consumption	Cost
DCPI Resin (PETA + Spiropyran SP/MC)	Medium (10)	Low	Medium	Medium 10-50 mW/cm^2	Low 10-20 \$/100g
Photoresist (PETA + DETC, ITX - based)	Relatively High (10^2 - 10^3)	Low	Medium	Relatively High 100-500 mW/cm^2	Medium 20-30 \$/100g
Rare - Earth Doped Nanomaterials ($NaYF_4$: Yb^{3+}/Tm^{3+})	Relatively High (10^2 - 10^3)	High	High	High 50-150 mW/cm^2	High 30-70 \$/10mg
Quantum Dots/QDs (CdSe@ZnS, Mn^{2+}-ZnSe)	High (over 10^3 under STED modulation)	High	Medium	Relatively High200-800 mW/cm^2	High 40-110 \$/10mg
Nanodiamond NV Centers	Extremely High (10^4)	Extremely High	Extremely High	Low 1-10 mW/cm^2	Extremely High 430-1130 \$/mg
